# MICROGLIA MAY INSTRUCT SYNAPTIC FATE VIA SIRPα IN MOUSE RETINA

**DOI:** 10.1093/geroni/igz038.3507

**Published:** 2019-11-08

**Authors:** Danye Jiang, Melanie Samuel

**Affiliations:** Baylor College of Medicine, Houston, Texas, United States

## Abstract

As we age, our nervous system undergoes many deleterious alterations: cognitive and sensory functions decrease while the risk of disease increases. Synapses are responsible for neural information processing, and the decline of these structures via microglia-mediated remodeling is thought to underline many age-related neural changes. However, the molecular pathways responsible for microglia-mediated synapses removal in development and old age remain unknown. To begin to elucidate these pathways, we leveraged the precisely organized murine retina where neurons form synapses in distinct lamina. Using this system, we screened 102 lacZ reporter lines available through the Knockout Mouse Project (KOMP) and uncovered a unique synapse regulatory candidate, SIRPα. We show that SIRPα is present in microglia prior to synapse formation but becomes selectively enriched in neural synapse terminals as these connections mature. Further, the levels of SIRPα decrease in the context of age-related neural decline. In ongoing studies, we are testing the hypothesis that neuronal SIRPα regulates its receptor CD47 to modulate refinement by microglial SIRPα. Together, these studies will resolve the molecular cues through which microglia prune synapses in development and dissect how these programs may go awry in the context of aging and disease.

